# Body Mass Index and All-Cause Mortality in a Large Prospective Cohort of White and Black U.S. Adults

**DOI:** 10.1371/journal.pone.0109153

**Published:** 2014-10-08

**Authors:** Alpa V. Patel, Janet S. Hildebrand, Susan M. Gapstur

**Affiliations:** Epidemiology Research Program, American Cancer Society, Atlanta, Georgia, United States of America; "Mario Negri" Institute for Pharmacological Research, Italy

## Abstract

Remaining controversies on the association between body mass index (BMI) and mortality include the effects of smoking and prevalent disease on the association, whether overweight is associated with higher mortality rates, differences in associations by race and the optimal age at which BMI predicts mortality. To assess the relative risk (RR) of mortality by BMI in Whites and Blacks among subgroups defined by smoking, prevalent disease, and age, 891,572 White and 38,119 Black men and women provided height, weight and other information when enrolled in the Cancer Prevention Study II in 1982. Over 28 years of follow-up, there were 434,400 deaths in Whites and 18,702 deaths in Blacks. Cox proportional-hazards regression was used to estimate multivariable-adjusted relative risks (RR) and 95% confidence intervals (CI). Smoking and prevalent disease status significantly modified the BMI-mortality relationship in Whites and Blacks; higher BMI was most strongly associated with higher risk of mortality among never smokers without prevalent disease. All levels of overweight and obesity were associated with a statistically significantly higher risk of mortality compared to the reference category (BMI 22.5–24.9 kg/m^2^), except among Black women where risk was elevated but not statistically significant in the lower end of overweight. Although absolute mortality rates were higher in Blacks than Whites within each BMI category, relative risks (RRs) were similar between race groups for both men and women (p-heterogeneity by race  = 0.20 for men and 0.23 for women). BMI was most strongly associated with mortality when reported before age 70 years. Results from this study demonstrate for the first time that the BMI-mortality relationship differs for men and women who smoke or have prevalent disease compared to healthy never-smokers. These findings further support recommendations for maintaining a BMI between 20–25 kg/m^2^ for optimal health and longevity.

## Introduction

Excess body weight is known to increase risk of premature mortality and various chronic diseases including cardiovascular disease, diabetes, and various types of cancer [1], [2]. Both the disease etiology implications and public health impact of high body mass index (BMI) are vitally important given the extremely high rates of overweight and obesity in the U.S. and worldwide. Numerous observational studies [2]–[5] have reported on the relationship between obesity and mortality with quantitatively and qualitatively varied results.

Despite the large number of studies to date, four major unresolved issues in the study of obesity and mortality still exist. The first two related issues include whether overweight (BMI 25–<30 kg/m^2^) is beneficial or detrimental in relation to total mortality and to what extent smoking and prevalent disease, conditions that both cause weight loss and shortened survival, influence the relationship between overweight and total mortality. A large pooled analysis that included 1.46 million Caucasian men and women found that smoking and prevalent disease significantly modified the BMI-mortality relationship. Among healthy, never smokers there was a statistically significant higher risk of mortality in underweight (BMI <18.5 kg/m^2^) and in the lowest end of normal weight (18.5–19.9 kg/m^2^) groups as well as overweight and obese individuals compared to the upper end of normal weight (22.5–24.9 kg/m^2^), and optimal BMI was between 20.0 and 24.9 kg/m^2^ [3]. Conversely, a recent meta-analysis [5] including 97 studies reported that overweight was associated with lower all-cause mortality compared to normal weight, and reported that exclusion of smokers and individuals with prevalent disease had little effect on the magnitude of risk estimates. Unlike the large pooled analysis, the meta-analysis by Flegal et al, examined associations relative to the full range of normal weight (reference category was 18.5–24.9 kg/m^2^). Inclusion of the low end of the normal weight in the reference group would result in mortality rates that are higher than if the reference group were limited to the apparently optimal range of 20–24.9 kg/m^2^, and would result in a lower observed relative risk in the overweight group, especially when smokers and individuals with prevalent disease are included.

The third issue is whether the association between BMI and mortality differs by race. While recent, large pooled analyses have examined the relationship between BMI and all-cause death rates in Caucasians [3] and Asians [6], the BMI-mortality dose-response relationship has not been well-characterized in African Americans. Among the relatively few studies on this issue in African Americans [4], [7]–[17], most [8], [10], [12]–[17] suggest that the association between BMI and mortality may be weaker in Blacks than Whites. However, studies of African Americans had relatively small sample sizes and/or short follow-up time. Finally, the fourth issue is that the appropriate age window of the exposure (the age at which body mass maximally predicts subsequent mortality) remains unclear. Results of various studies suggest that BMI measured at younger ages is more predictive of subsequent mortality long term because it is more effective in measuring adiposity and accounting for pre-existing disease than BMI measured later in life [4], [8], [9], [18]–[21].

The American Cancer Society's Cancer Prevention Study II is well-suited to the study of BMI in relation to mortality, due to its prospective design, large size of both Black and White men and women, and long follow-up period. Our initial paper examining the association between BMI and total mortality was published in 1999 when the cohort had been followed for 14 years [4]. That analysis was based on 14-years of follow-up and had a limited number deaths among Black men and women, especially among those who were obese (n = 83 deaths among men, n = 319 deaths among women). Here, we present results on the four issues described above with follow-up extended to 28 years including over 450,000 deaths.

## Methods

### Study Population

Subjects for this analysis were selected from the 1,184,387 participants (508,227 men and 676,160 women) in the Cancer Prevention Study II, a prospective study of mortality among men and women in the United States begun by the American Cancer Society in 1982 [22]. Participants were identified and enrolled by more than 77,000 volunteers in all 50 states, the District of Columbia, and Puerto Rico. Families were enrolled if at least one household member was 45 years of age or older, and all enrolled members were at least 30 years old. The average age of participants at enrollment was 57 years old. In 1982, participants completed a confidential questionnaire, through which they provided information on demographic characteristics, personal and family history of cancer and other diseases, environmental and occupational exposures, and factors related to lifestyle, behavior and diet. All aspects of CPS-II have been reviewed and approved by the Emory University Institutional Review Board, and all data were de-identified prior to analysis.

Participants were asked to provide their current weight, weight one year prior to enrollment, and height (without shoes). Subjects with missing values for height or current weight were excluded from the analysis (N = 29,320), as were those with extreme values (99.9^th^ percentile) for height or weight or extreme underweight (BMI <15) (N = 6,484). In order to adequately control for smoking, we excluded subjects with missing data for the smoking questions (N = 46,900), smokers with no information on number of cigarettes per day (N = 87,342), former smokers with no information on years since quitting (N = 2,080), and smokers whose smoking status at baseline (current or former) was unknown (N = 20,068). We also excluded men who had smoked only pipes or cigars but not cigarettes (N = 40,462) because their amount of smoking was not well defined; pipe and cigar smoking was not queried in women. Finally, we excluded all races other than White or Black (N = 21,860). The remaining eligible population, after exclusions, consisted of 341,196 White men (194,588 deaths), 12,559 Black men (7,368 deaths), 550,556 White women (239,812 deaths), and 25,560 Black women (11,334 deaths).

### Classifying Body-Mass Index

Body-mass index (BMI) (kg/m^2^) was classified as follows: 15.0 to 18.4, 18.5 to 19.9; 20.0 to 22.4; 22.5 to 24.9 (reference group); 25.0 to 27.4; 27.5 to 29.9; 30.0 to 34.9; 35.0 to 39.9; 40.0 and higher. Due to small numbers, the highest category examined for Black men was 35.0 and higher. This categorization scheme includes finer groupings of the World Health Organization (WHO) definitions of “underweight” as body mass index (BMI) less than 18.5 kg/m^2^, the “normal” range 18.5 to 24.9 kg/m^2^, “overweight” range 25.0 to 29.9 kg/m^2^, and “obese” as greater than or equal to 30.0 kg/m^2^ [23]. Additionally, these finer groupings were selected *a priori* for consistency with several previously published studies including the large pooled study of 1.46 million White participants reporting the nadir of the dose-response curve was at BMI 22.5 to 24.9 [3].

### End Points

Deaths occurring between the months of enrollment and December 31, 2010, were ascertained through personal inquiries by volunteers in September 1984, September 1986, and September 1988, and thereafter through linkage with the National Death Index (NDI) [24]. As of December 31, 2010, 50.5 percent of the participants had died and 49.5 percent were still living; 0.3 percent were lost to follow-up on September 1, 1988 due to insufficient data for linkage with the NDI. Multiple cause-of-death codes have been obtained for 99.3 percent of all deaths.

The primary end point in this analysis was deaths from all causes. In secondary analyses, deaths from all cardiovascular disease (ICD-9 codes 390 through 459; ICD-10 codes I00 through I99), all cancer (ICD-9 codes 140 through 208; ICD-10 codes C00 through C97), and all other causes, were examined separately in relation to BMI.

### Statistical Analysis

Age-adjusted mortality rates according to BMI in men and women, race-specific, were calculated by direct standardization to the age distribution of the CPS-II male and female populations using 5-year age categories. Multivariable-adjusted relative risks (RRs) and 95% confidence intervals (CIs) were estimated in Cox proportional-hazards regression models stratifying on single year of age at enrollment. Models simultaneously adjusted for education (less than high school, high school graduate, some college/vocational training, college graduate and higher), physical activity (none, slight, moderate, or heavy exercise), alcohol use (nondrinker, <1 drink per day, 1 drink per day, >1 drink daily), marital status (married vs. single, widowed, or divorced), aspirin use (yes/no), fat consumption, vegetable consumption (sex-specific tertiles), and estrogen replacement therapy among women (yes/no). Models that included ever smokers were also adjusted for smoking status, frequency and time since quitting (current smoker with cigarettes per day (cpd) categorized as ≤10, 11–20, 21–30, 31–40, or 40 or more; former smoker having quit within the last year and smoked ≤10, 11–20, 21–30, 31–40, or 40 or more cpd; former smoker having quit 1 to 9 years prior and smoked ≤10, 11–20, 21–30, 31–40, or 40 or more cpd; former smoker having quit 10–19 years prior and smoked ≤10, 11–20, 21–30, 31–40, 40 or more cpd, and former smoker having quit at least 20 years prior and smoked ≤10, 11–20, 21–30, 31–40, or 40 or more cpd) and those that included prevalent disease also adjusted for disease status (no/yes for cancer, heart disease, stroke, or emphysema; yes for chronic bronchitis or asthma).

We examined the association between BMI and all-cause mortality for each of four mutually exclusive race and gender-specific subgroups according to smoking status [never, ever] and prevalent disease status [yes/no] defined as a history of cancer (except non-melanoma skin cancer), heart disease, stroke, respiratory diseases (chronic bronchitis, emphysema, or asthma), current illness of any kind, or weight loss of 10 or more lbs (4.5 kg) in the previous year. Effect modification by smoking and prevalent disease combined (healthy never smokers, healthy smokers, never smokers with prevalent disease, smokers with prevalent disease) and by race were evaluated in multivariate models using the likelihood ratio test and a p-value <0.05 was considered statistically significant. In Black and Whites combined, we examined the association between BMI and mortality by age at enrollment (younger than 50, 50–59, 60–69, 70 years or older) but this analysis was limited to the healthy never smokers because smoking and prevalent disease significantly modified the association between BMI and mortality. Finally, we examined the associations of BMI with cardiovascular disease and cancer mortality separately.

## Results

The mean age at baseline was 56 years old for White men, 55 for Black men, 57 for White women and 55 for Black women. The mean BMI was 25.8, 26.8, 24.6, 27.1 kg/m^2^ for White and Black men and women, respectively. Selected baseline characteristics for each sex-race group by BMI category are presented in Table 1. BMI was inversely related to age at baseline for all groups except Black women. A strong positive relationship was observed between BMI and the prevalence of never smokers in both men and women at the time of enrollment, regardless of race. In contrast, there was a curvilinear relationship between BMI and prevalent disease, such that individuals at both high and low extremes of BMI were more likely to report disease at baseline than persons within the WHO normal range of BMI (18.5–24.9 kg/m^2^). For all groups, BMI was also inversely related to educational status at BMI levels of 22.5 kg/m^2^ or greater.

**Table 1:** Baseline characteristics of all CPS-II men and women by race and BMI (kg/m^2^).

Characteristic	BMI (kg/m^2^)								
	15.0–18.4	18.5–19.9	20.0–22.4	22.5–24.9	25.0–27.4	27.5–29.9	30.0–34.9	35.0–39.9	≥40.0
**MEN**									
**White Race (Total N = 341,196; Deaths = 194,588)**									
Number of men	2,535	5,115	36,781	93,990	115,561	53,776	29,324	3,744	370
Number of deaths	1,940	3,609	22,402	52,358	63,552	30,453	17,615	2,415	244
Mean Age (yrs)	62	60	58	58	57	56	55	54	53
≥High school (%)^a^	55.6	58.7	66.3	67.8	64.0	58.8	56.4	53.8	51.3
Married (%)^a^	89.5	90.0	92.2	94.3	95.4	95.4	94.9	93.8	87.4
Nondrinker (%)^a^	19.8	21.1	18.7	17.3	16.6	17.1	18.3	21.2	24.4
Never smoker (%)^a^	24.0	26.6	33.0	34.8	34.1	33.5	34.2	34.4	32.4
Prevalent diseases (%)^a^	50.4	42.4	33.8	31.7	32.6	34.9	37.6	42.5	41.4
**Black Race (Total N = 12,559; Deaths = 7,368)**									
Number of men	119	250	1,368	2,799	3,807	2,289	1,624	264	39
Number of deaths	82	180	818	1,611	2,200	1,319	977	153	28
Mean Age (yrs)	59	57	55	56	55	55	54	52	52
≥High school (%)^a^	48.6	38.9	51.6	54.6	53.0	52.6	45.9	38.7	37.4
Married (%)^a^	75.4	75.2	79.1	84.3	84.6	87.9	85.6	85.5	88.5
Nondrinker (%)^a^	16.1	14.7	13.7	15.5	16.2	17.1	16.9	17.6	20.2
Never smoker (%)^a^	26.6	17.9	24.4	31.6	33.7	38.3	43.9	44.9	42.4
Prevalent diseases (%)^a^	44.9	40.9	32.2	29.2	31.9	34.2	40.1	41.6	48.8
**WOMEN**									
**White Race (Total N = 550,556; Deaths = 239,812)**									
Number of women	14,076	41,470	148,270	144,815	98,373	46,003	43,610	10,738	3,201
Number of deaths	7,681	16,271	56,019	61,462	46,167	22,870	22,033	5,536	1,773
Mean Age (yrs)	58	55	55	57	58	58	57	54	53
≥High school (%)^a^	61.0	64.0	61.9	56.1	52.0	48.8	46.7	45.3	43.1
Married (%)^a^	69.8	74.4	77.9	79.0	78.3	76.7	74.8	72.3	67.3
Nondrinker (%)^a^	21.6	19.7	19.2	20.4	22.0	24.0	25.8	28.8	28.9
Never smoker (%)^a^	45.1	50.0	53.9	57.8	61.7	64.2	66.1	65.0	64.3
Prevalent diseases (%)^a^	37.2	30.7	29.7	32.2	35.2	38.6	42.1	47.9	50.6
**Black Race (Total N = 25,560; Deaths = 11,334)**									
Number of women	403	779	3,324	5,295	5,757	3,603	4,513	1,345	541
Number of deaths	214	333	1,285	2,032	2,497	1,706	2,256	698	313
Mean Age (yrs)	56	54	54	54	56	56	56	54	54
≥High school (%)^a^	55.4	60.4	62.2	59.5	54.5	50.6	46.0	42.0	38.1
Married (%)^a^	50.9	51.2	58.3	62.1	61.9	62.3	59.2	54.5	51.8
Nondrinker (%)^a^	19.7	18.9	19.4	20.1	20.8	22.0	21.9	23.5	27.2
Never smoker (%)^a^	42.3	46.6	50.0	55.8	59.5	63.3	66.0	65.7	67.5
Prevalent diseases (%)^a^	40.8	31.0	29.6	31.4	35.2	36.6	41.7	46.7	51.7

a Adjusted to the age distribution of the CPS-II male/female population.

Tables 2 and 3 present the BMI-mortality relationship by race for men and women, respectively, stratified by smoking and prevalent disease status. Smoking and prevalent disease modified the association between BMI and mortality in all four groups (interaction p = 2.4×10^−39^ for White men, p = 0.055 for Black men, p = 1.6×10^−32^ for White women, and p = 0.02 for Black women). For White men and women, regardless of smoking status or prevalent disease, underweight was associated with higher risk of mortality compared to BMI 22.5–24.9 kg/m^2^. For Black men, underweight was associated with higher risk of mortality in those with prevalent disease, regardless of smoking status, but not in Black men without prevalent disease. In Black women, underweight was associated with higher mortality in all groups, albeit a non-statistically significant elevated risk for the group defined as never smokers with prevalent disease. Among all underweight BMI categories, age-standardized death rates were highest in men and women who smoked and had prevalent disease, and rates were lowest in healthy never smokers.

**Table 2:** Rates and relative risks of death from any cause among men according to BMI, smoking, prevalent disease status and race, CPS-II 1982-2010.

Group	BMI								
	15.0–18.4	18.5–19.9	20.0–22.4	22.5–24.9	25.0–27.4	27.5–29.9	30.0–34.9	35.0–39.9	≥40.0
**White smoker with prevalent disease** a									
No. of deaths	1,038	1,538	7,427	15,612	18,706	9,273	5,647	828	80
Age-stand. rate^b^	8258	6759	5045	4048	3843	4095	4662	5357	6326
Multivariate RR^c^	1.66	1.47	1.18	1.00	0.96	1.02	1.15	1.28	1.52
95% CI	(1.55–1.76)	(1.39–1.55)	(1.15–1.22)	-	(0.94–0.98)	(1.00–1.05)	(1.12–1.19)	(1.19–1.37)	(1.22–1.89)
Stand. rate difference	4210	2711	997	0	−206	46	614	1308	2278
**White smoker without prevalent disease** d									
No. of deaths	500	1,217	8,898	21,389	26,250	12,248	6,710	833	92
Age-stand. rate^b^	3633	3682	2977	2547	2522	2702	3125	3760	4954
Multivariate RR^c^	1.29	1.34	1.15	1.00	0.99	1.06	1.24	1.50	2.00
95% CI	(1.18–1.42)	(1.27–1.42)	(1.12–1.18)	-	(0.97–1.01)	(1.04–1.08)	(1.21–1.28)	(1.40–1.60)	(1.63–2.45)
Stand. rate difference	1086	1134	429	0	−26	154	578	1213	2407
**White never smoker with prevalent disease** a									
No. of deaths	211	415	2,415	5,271	6,383	3,160	1,947	302	19
Age-stand. rate^b^	4767	4726	3131	2657	2741	2925	3550	4323	3128
Multivariate RR^c^	1.34	1.38	1.11	1.00	1.06	1.14	1.42	1.75	1.18
95% CI	(1.16–1.54)	(1.25–1.53)	(1.05–1.16)	-	(1.02–1.10)	(1.09–1.19)	(1.35–1.50)	(1.55–1.97)	(0.75–1.86)
Stand. rate difference	2110	2068	473	0	83	267	893	1666	470
**White never smoker without prevalent disease** d									
No. of deaths	191	439	3,662	10,086	12,213	5,772	3,311	452	53
Age-stand. rate^b^	2358	2015	1759	1691	1776	2038	2364	3145	3965
Multivariate RR^c^	1.25	1.06	1.01	1.00	1.07	1.28	1.50	2.17	2.52
95% CI	(1.08–1.45)	(0.96–1.17)	(0.97–1.05)	-	(1.04–1.10)	(1.24–1.32)	(1.44–1.56)	(1.98–2.39)	(1.92–3.30)
Stand. rate difference	667	324	68	0	85	347	673	1454	2274

	BMI							
	15.0–18.4	18.5–19.9	20.0–22.4	22.5–24.9	25.0–27.4	27.5–29.9	30.0–34.9	≥35.0
**Black smoker with prevalent disease** a								
No. of deaths	39	67	261	439	627	369	284	57
Age-stand. rate^b^	9346	6692	5451	4901	4667	4715	4985	5184
Multivariate RR^c^	1.35	1.36	1.10	1.00	0.97	0.95	1.06	1.14
95% CI	(0.95–1.93)	(1.04–1.77)	(0.94–1.29)	-	(0.86–1.11)	(0.83–1.10)	(0.90–1.23)	(0.86–1.52)
Stand. rate difference	4445	1792	551	0	−234	−185	85	284
**Black smoker without prevalent disease** d								
No. of deaths	24	78	390	732	954	494	299	58
Age-stand. rate^b^	3090	4520	3652	3061	3190	3075	3386	3189
Multivariate RR^c^	0.99	1.57	1.14	1.00	1.03	1.00	1.11	1.15
95% CI	(0.66–1.50)	(1.24–2.00)	(1.00–1.29)	-	(0.94–1.14)	(0.89–1.12)	(0.97–1.28)	(0.88–1.51)
Stand. rate difference	30	1459	591	0	129	14	325	129
**Black never smoker with prevalent disease** a								
No. of deaths	15	18	65	158	201	166	159	24
Age-stand. rate^b^	7523	7485	4548	3693	2989	3278	3801	3498
Multivariate RR^c^	1.76	2.00	1.36	1.00	0.76	0.88	1.00	0.91
95% CI	(0.98–3.17)	(1.17–3.45)	(0.99–1.86)	-	(0.60–0.95)	(0.70–1.12)	(0.79–1.27)	(0.57–1.46)
Stand. rate difference	3830	3793	855	0	−704	−415	108	−195
**Black never smoker without prevalent disease** d								
No. of deaths	4	17	102	282	418	290	235	42
Age-stand. rate^b^	1398	3448	2069	1919	2150	2307	2735	3316
Multivariate RR^c^	0.78	1.60	1.05	1.00	1.18	1.24	1.53	1.97
95% CI	(0.29–2.13)	(0.94–2.72)	(0.83–1.34)	-	(1.01–1.38)	(1.05–1.47)	(1.28–1.83)	(1.41–2.76)
Stand. rate difference	−520	1529	151	0	232	388	817	1398

a One or more of the following conditions was reported at study entry: prevalent cancer (except non melanoma skin), heart disease, stroke, respiratory disease (chronic bronchitis, emphysema, asthma), currently sick, or weight loss of ≥10 lbs. in past year.

b Rate per 100,000 standardized to the age-distribution of the CPS-II men.

c Cox proportional hazards model, adjusted for age, race, education, physical activity, alcohol use, marital status, aspirin use, fat consumption, and vegetable consumption

d None of the conditions listed in footnote(a) were reported.

**Table 3:** Relative risks of death from any cause among women according to BMI, smoking, prevalent disease status and race, CPS-II 1982–2010.

Group	BMI								
	15.0–18.4	18.5–19.9	20.0–22.4	22.5–24.9	25.0–27.4	27.5–29.9	30.0–34.9	35.0–39.9	≥40.0
**White smoker with prevalent disease** a									
No. of deaths	2,045	3,499	10,721	11,099	7,949	3,963	3,966	1,135	404
Age-stand. rate^b^	4525	3071	2630	2565	2742	2972	3154	3496	4102
Multivariate RR^c^	1.69	1.21	1.05	1.00	1.04	1.12	1.20	1.32	1.58
95% CI	(1.61–1.77)	(1.16–1.26)	(1.03–1.08)	-	(1.01–1.08)	(1.08–1.17)	(1.16–1.25)	(1.24–1.40)	(1.43–1.74)
Stand. rate difference	1960	506	65	0	176	407	588	931	1536
**White smoker without prevalent disease** d									
No. of deaths	2,167	5,161	16,863	15,393	9,624	4,126	3,509	884	271
Age-stand. rate^b^	2751	1941	1735	1761	1905	2040	2259	2640	3232
Multivariate RR^c^	1.56	1.14	1.02	1.00	1.06	1.13	1.26	1.53	1.92
95% CI	(1.49–1.63)	(1.10–1.18)	(1.00–1.05)	-	(1.03–1.08)	(1.09–1.17)	(1.21–1.31)	(1.43–1.64)	(1.70–2.16)
Stand. rate difference	990	180	−26	0	144	279	498	879	1472
**White never smoker with prevalent disease** a									
No. of deaths	1,783	3,104	10,748	13,546	11,453	6,440	6,568	1,798	565
Age-stand. rate^b^	3402	2366	2044	2047	2165	2351	2521	2901	3361
Multivariate RR^c^	1.44	1.10	1.01	1.00	1.05	1.13	1.25	1.51	1.82
95% CI	(1.37–1.52)	(1.06–1.14)	(0.98–1.03)	-	(1.02–1.07)	(1.10–1.17)	(1.21–1.28)	(1.44–1.59)	(1.67–1.98)
Stand. rate difference	1355	319	−3	0	118	304	475	854	1315
**White never smoker without prevalent disease** d									
No. of deaths	1,686	4,507	17,687	21,424	17,141	8,341	7,990	1,719	533
Age-stand. rate^b^	1765	1392	1280	1363	1514	1654	1849	2178	2798
Multivariate RR^c^	1.20	1.04	0.97	1.00	1.09	1.21	1.39	1.79	2.45
95% CI	(1.14–1.26)	(1.01–1.07)	(0.95–0.99)	-	(1.07–1.11)	(1.18–1.24)	(1.35–1.43)	(1.70–1.88)	(2.25–2.67)
Stand. rate difference	402	29	−83	0	151	291	486	815	1436

	BMI								
	15.0–18.4	18.5–19.9	20.0–22.4	22.5–24.9	25.0–27.4	27.5–29.9	30.0–34.9	35.0–39.9	≥40.0
**Black smoker with prevalent disease** a									
No. of deaths	60	70	268	386	445	287	377	131	73
Age-stand. rate^b^	3919	3831	3203	2791	2783	3209	3392	3359	4473
Multivariate RR^c^	1.40	1.52	1.17	1.00	0.99	1.10	1.12	1.14	1.43
95% CI	(1.06–1.84)	(1.17–1.98)	(1.00–1.38)	-	(0.86–1.13)	(0.94–1.29)	(0.97–1.30)	(0.93–1.40)	(1.10–1.86)
Stand. rate difference	1128	1040	412	0	−8	417	601	568	1682
**Black smoker without prevalent disease** d									
No. of deaths	57	109	399	525	558	320	390	109	30
Age-stand. rate^b^	3328	2433	1975	1751	2026	2064	2492	2697	2630
Multivariate RR^c^	2.11	1.42	1.12	1.00	1.13	1.18	1.36	1.52	1.42
95% CI	(1.59–2.80)	(1.15–1.76)	(0.98–1.28)	-	(1.00–1.28)	(1.02–1.36)	(1.19–1.55)	(1.23–1.88)	(0.98–2.07)
Stand. rate difference	1577	682	224	0	275	313	741	946	878
**Black never smoker with prevalent disease** a									
No. of deaths	45	68	255	431	620	448	688	238	114
Age-stand. rate^b^	3908	2530	2554	2255	2434	2372	2510	3024	3893
Multivariate RR^c^	1.11	1.06	1.14	1.00	1.15	1.14	1.19	1.45	2.06
95% CI	(0.79–1.55)	(0.81–1.38)	(0.98–1.34)	-	(1.01–1.30)	(0.99–1.30)	(1.05–1.34)	(1.23–1.70)	(1.67–2.56)
Stand. rate difference	1653	275	299	0	179	117	255	769	1638
**Black never smoker without prevalent disease** d									
No. of deaths	52	86	363	690	874	651	801	220	96
Age-stand. rate^b^	2224	1697	1418	1479	1572	1854	1973	2298	2566
Multivariate RR^c^	1.38	1.04	0.97	1.00	1.08	1.27	1.32	1.64	1.78
95% CI	(1.03–1.85)	(0.82–1.30)	(0.85–1.10)	-	(0.97–1.19)	(1.14–1.42)	(1.19–1.46)	(1.40–1.91)	(1.43–2.21)
Stand. rate difference	744	218	−61	0	93	375	494	819	1087

a One or more of the following conditions was reported at study entry: prevalent cancer (except non melanoma skin), heart disease, stroke, respiratory disease (chronic bronchitis, emphysema, asthma), currently sick, or weight loss of ≥10 lbs. in past year.

b Rate per 100,000 standardized to the age-distribution of the CPS-II women.

c Cox proportional hazards model, adjusted for age, race, education, physical activity, alcohol use, marital status, aspirin use, fat consumption, vegetable consumption, and postmenopausal estrogen use

d None of the conditions listed in footnote(a) were reported.

Relative risks were higher for overweight and obese compared to the upper end of normal weight (BMI 22.5–24.9 kg/m^2^) among the healthy never smokers compared to those who smoked and/or had prevalent disease (Tables 2 and 3). In this group of never smokers without prevalent disease, among both men and women, the RR's for overweight Blacks appear to be similar in magnitude to those for Whites whereas at the highest levels of BMI, the RR's for Blacks appear to be marginally lower compared to those for Whites. For example, among healthy never smokers, the multivariable-adjusted RR for White women with BMI> = 40 kg/m^2^ was 2.45 (95% CI 2.25–2.67) whereas it was 1.78 for Black women (95% CI 1.43–2.21). However, there was no evidence of statistical interaction by race (interaction p = 0.23 for women and p = 0.20 for men).

Due to the lack of effect modification by race and the profound modifying effect of smoking and prevalent disease on the association between BMI and mortality, analyses stratified by age and grouped cause of death are presented among never smokers without prevalent disease in both races combined. The magnitude of the association between BMI and total mortality varied substantially by the age at which BMI was reported (Table 4). BMI at ages younger than 70 years was much more strongly associated with risk of death in both men and women than was BMI at older ages. For BMI at ages less than 60 years and 60–69 years, all categories of overweight and obesity were associated with higher risk of mortality. The association between BMI and mortality was greatly attenuated when BMI was reported at age 70 and above; however, in women, associations remained statistically significant for all categories of BMI>27.4 kg/m^2^. Overweight and obesity were associated with all grouped causes of death, but were generally stronger for death from cardiovascular disease and other causes compared to cancer for both men and women (Table 5).

**Table 4:** Relative risks of death from any cause according to BMI and age at enrollment among men and women who are never smokers without prevalent disease, CPS-II 1982–2010.

Group	BMI								
	15.0–18.4	18.5–19.9	20.0–22.4	22.5–24.9	25.0–27.4	27.5–29.9	30.0–34.9	35.0–39.9	≥40.0
**MEN**									
**Age <60 years**									
No. of deaths	46	91	1,001	3,093	4,376	2,506	1,760	305	41
Age-stand. rate^a^	1313	1070	1044	1073	1222	1535	2005	2907	4270
Multivariate RR^b^	1.21	1.00	0.99	1.00	1.13	1.39	1.84	2.63	3.97
95% CI	(0.90–1.61)	(0.81––1.23)	(0.92–1.06)	-	(1.08–1.18)	(1.32–1.46)	(1.73–1.95)	(2.33–2.96)	(2.91–5.41)
Stand. rate difference	240	−3	−29	0	149	462	932	1834	3198
**Age 60–69 years**									
No. of deaths	75	169	1,536	4,454	5,587	2,568	1,345	142	19
Age-stand. rate^a^	2400	2083	1778	1780	1928	2278	2471	3178	4753
Multivariate RR^b^	1.51	1.17	0.99	1.00	1.08	1.29	1.40	1.82	3.15
95% CI	(1.20–1.89)	(1.01–1.37)	(0.94–1.05)	-	(1.04–1.12)	(1.23–1.36)	(1.32–1.49)	(1.54–2.16)	(2.01–4.95)
Stand. rate difference	619	302	−3	0	148	498	691	1398	2972
	**15.0–18.4**	**18.5–19.9**	**20.0–22.4**	**22.5–24.9**	**25.0–27.4**	**27.5–29.9**	**30.0–34.9**	**≥35.0**	
**Age ≥70 years**									
No. of deaths	74	196	1,227	2,821	2,668	988	441	40	
Age-stand. rate^a^	2751	2295	2349	2275	2302	2400	2334	2485	
Multivariate RR^b^	1.01	1.01	1.03	1.00	1.00	1.08	1.06	1.15	
95% CI	(0.80–1.28)	(0.87–1.17)	(0.96–1.10)	-	(0.94–1.05)	(1.01–1.16)	(0.95–1.17)	(0.84–1.57)	
Stand. rate difference	475	19	73	0	26	124	58	209	

	BMI								
	15.0–18.4	18.5–19.9	20.0–22.4	22.5–24.9	25.0–27.4	27.5–29.9	30.0–34.9	35.0–39.9	≥40.0
**WOMEN**									
**Age <60 years**									
No. of deaths	372	1,307	5,214	5,704	4,658	2,544	2,949	897	346
Age-stand. rate^a^	966	768	740	802	978	1138	1378	1893	2556
Multivariate RR^b^	1.18	0.96	0.93	1.00	1.19	1.37	1.66	2.28	3.31
95% CI	(1.06–1.31)	(0.91–1.02)	(0.89–0.96)	-	(1.14–1.24)	(1.31–1.44)	(1.59–1.74)	(2.12–2.44)	(2.97–3.69)
Stand. rate difference	164	−34	−63	0	175	336	576	1091	1754
**Age 60–69 years**									
No. of deaths	571	1,681	6,931	9,001	7,665	3,934	3,699	708	204
Age-stand. rate^a^	1571	1416	1297	1353	1479	1659	1850	2090	2455
Multivariate RR^b^	1.16	1.08	0.97	1.00	1.08	1.23	1.37	1.59	1.94
95% CI	(1.07–1.27)	(1.03–1.14)	(0.94–1.00)	-	(1.05–1.12)	(1.18–1.27)	(1.32–1.43)	(1.47–1.71)	(1.69–2.23)
Stand. rate difference	218	63	−56	0	125	306	497	737	1102
**Age ≥70 years**									
No. of deaths	795	1,605	5,905	7,409	5,692	2,514	2,143	334	79
Age-stand. rate^a^	2404	2050	1937	1895	1946	2038	2129	2348	2563
Multivariate RR^b^	1.24	1.07	1.02	1.00	1.02	1.07	1.13	1.33	1.29
95% CI	(1.15–1.33)	(1.02–1.13)	(0.98–1.05)	-	(0.99–1.06)	(1.02–1.12)	(1.08–1.19)	(1.19–1.49)	(1.03–1.61)
Stand. rate difference	509	155	43	0	51	143	234	453	668

a Rate per 100,000 standardized to the age-distribution of the CPS-II men/women.

b Cox proportional hazards model, adjusted for age, race, education, physical activity, alcohol use, marital status, aspirin use, fat consumption, vegetable consumption, and postmenopausal estrogen use (women)

**Table 5:** Relative risk of death from cardiovascular, cancer, or other causes according to BMI among men and women who are never smokers without prevalent disease, CPS-II 1982–2010.

Group	BMI								
	15.0–18.4	18.5–19.9	20.0–22.4	22.5–24.9	25.0–27.4	27.5–29.9	30.0–34.9	35.0–39.9	≥40.0
**MEN**									
**Cardiovascular disease death**									
No. of deaths	72	164	1,194	3,380	4,331	2,143	1,298	193	22
Age-stand. rate^a^	864	737	559	554	613	723	866	1218	1272
Multivariate RR^b^	1.23	1.05	0.95	1.00	1.15	1.42	1.74	2.73	2.89
95% CI	(0.97–1.55)	(0.90–1.23)	(0.89–1.02)	-	(1.10–1.20)	(1.34–1.50)	(1.63–1.85)	(2.36–3.16)	(1.90–4.41)
Stand. rate difference	310	183	5	0	59	169	312	664	718
	**15.0–18.4**	**18.5–19.9**	**20.0–22.4**	**22.5–24.9**	**25.0–27.4**	**27.5–29.9**	**30.0–34.9**	**≥35.0**	
**Cancer death**									
No. of deaths	32	87	710	2,082	2,611	1,171	696	80	
Age-stand. rate^a^	390	397	341	348	373	392	454	431	
Multivariate RR^b^	1.01	1.09	0.96	1.00	1.08	1.16	1.36	1.38	
95% CI	(0.70–1.44)	(0.88–1.35)	(0.88–1.05)	-	(1.02–1.15)	(1.08–1.25)	(1.25–1.49)	(1.11–1.73)	
Stand. rate difference	42	49	−6	0	25	44	106	83	
	**15.0–18.4**	**18.5–19.9**	**20.0–22.4**	**22.5–24.9**	**25.0–27.4**	**27.5–29.9**	**30.0–34.9**	**35.0–39.9**	**≥40.0**
**All other causes of death**									
No. of deaths	67	133	1,156	2,607	2,934	1,354	756	123	22
Age-stand. rate^a^	790	602	541	426	414	458	505	782	1558
Multivariate RR^b^	1.57	1.15	1.21	1.00	1.00	1.15	1.29	2.21	3.66
95% CI	(1.23–2.01)	(0.96–1.37)	(1.13–1.30)	-	(0.95–1.06)	(1.08–1.23)	(1.19–1.40)	(1.84–2.65)	(2.40–5.59)
Stand. rate difference	364	177	116	0	−11	32	80	356	1132

	BMI								
	15.0–18.4	18.5–19.9	20.0–22.4	22.5–24.9	25.0–27.4	27.5–29.9	30.0–34.9	35.0–39.9	≥40.0
**WOMEN**									
**Cardiovascular disease death**									
No. of deaths	669	1,413	5,579	7,253	6,131	3,172	3,063	685	201
Age-stand. rate^a^	679	435	398	444	510	583	647	775	903
Multivariate RR^b^	1.26	0.99	0.95	1.00	1.11	1.28	1.46	2.06	2.39
95% CI	(1.16–1.36)	(0.93–1.05)	(0.92–0.98)	-	(1.07–1.15)	(1.23–1.34)	(1.40–1.52)	(1.90–2.22)	(2.08–2.76)
Stand. rate difference	235	−9	−46	0	66	139	203	331	459
**Cancer death**									
No. of deaths	246	864	3,316	4,158	3,321	1,696	1,690	365	130
Age-stand. rate^a^	255	252	232	268	299	330	363	395	521
Multivariate RR^b^	0.93	0.97	0.90	1.00	1.08	1.20	1.33	1.48	1.97
95% CI	(0.82–1.06)	(0.90–1.05)	(0.86–0.94)	-	(1.04–1.14)	(1.13–1.27)	(1.25–1.41)	(1.33–1.65)	(1.65–2.34)
Stand. rate difference	−13	−16	−36	0	31	62	95	127	253
**All other causes of death**									
No. of deaths	520	1,247	4,621	5,459	4,312	2,096	2,037	450	160
Age-stand. rate^a^	533	379	329	336	361	389	431	507	686
Multivariate RR^b^	1.42	1.16	1.03	1.00	1.04	1.13	1.28	1.69	2.41
95% CI	(1.30–1.56)	(1.09–1.23)	(0.99–1.07)	-	(1.00–1.09)	(1.07–1.19)	(1.22–1.35)	(1.53–1.86)	(2.06–2.82)
Stand. rate difference	197	43	−8	0	25	52	95	171	350

a Rate per 100,000 standardized to the age-distribution of the CPS-II men/women.

b Cox proportional hazards model, adjusted for age, race, education, physical activity, alcohol use, marital status, aspirin use, fat consumption, vegetable consumption, and postmenopausal estrogen use (women).

## Discussion

In this large prospective study of approximately one million Black and White men and women, we were able to address four major unresolved issues in the study of BMI and mortality. Results showed that men and women who were underweight were at higher risk of mortality as were men and women who were overweight and obese compared to normal weight men and women, and that smoking and prevalent disease significantly modified the association between BMI and mortality, such that the strongest associations were among never smokers without prevalent disease for men and women. In healthy never smokers, mortality rates were lowest within the upper end of the normal BMI category (i.e., 22.5–24.9 kg/m^2^) for all race sex groups. Although the results of our study showed no statistically significant differences in associations between BMI and mortality by race, overweight and obesity were associated with subsequent mortality among smokers and/or those with prevalent disease in White men, White women and Black women but not in in Black men. In addition, weight in late middle age but not older (i.e., 70 years or older) was strongly associated with future mortality.

There is growing evidence that residual confounding by smoking and/or reverse causality by prevalent disease attenuated the association between BMI and risk of mortality. Indeed, studies that included smokers (who tend to have a lower BMI) and individuals with diseases that cause weight loss generally show weaker associations with high BMI levels, and stronger associations with low BMI levels than do studies that exclude these subjects [4], [8], [25]–[28]. While controlling for smoking history can reduce its confounding effects, eliminating the residual effects of current smoking is essential for clarifying associations of BMI with mortality, and can only be achieved through exclusion of current smokers. On the other hand, prevalent disease exclusions may be more dependent on the characteristics and age of the study population. Consistent with the largest pooled analysis to date [3], in our study underweight and the lower end of the normal weight range were associated with higher risk of mortality among those who smoked or had prevalent disease.

Numerous studies have reported on the association between BMI and mortality in Caucasians [2]–[4], [8], [9], [18]–[21], [25]–[47]. In contrast, few studies have reported on the BMI-mortality relationship among Black men and women [4], [7]–[17], and among those studies, results are inconsistent with most studies showing a stronger association in Whites than in Blacks [8], [10], [12]–[17]. In fact, only two previous studies examined the BMI-mortality association in African American among healthy never-smokers. The first study included only women [11] and found that BMI-mortality associations were similar in magnitude as those reported in other studies among Whites; however, they were unable to directly compare results between Whites and Blacks as the study population included only Black women. The second was the earlier CPS-II analysis which included both men and women and was based on 14-years of follow-up [4]. Prior to the present analysis, that was the largest study to date among African Americans, and included a total of 485 deaths in Black men and 1,188 deaths in Black women. In that analysis, there was an excess risk of mortality with higher BMI in all race-sex groups, but the association between BMI and mortality appeared to be weaker in African Americans that in Whites. However, there were many fewer deaths identified among obese Black men and women (83 and 319 deaths, respectively) in the 14 year follow-up than in this study (247 and 1,002 deaths, respectively). In the current analysis, while Blacks had higher absolute mortality rates, the relative risks associated with BMI did not differ by race for men or women. The increased statistical power and precision is likely to have resulted in more stable estimates in the current compared to earlier analysis. In addition, the longer follow-up time would likely reduce confounding by undiagnosed disease-related weight loss.

The present study showed that the BMI-mortality association is stronger when BMI is assessed at younger ages (i.e. <70 years old in this study) in both men and women. Disease-related weight loss is less common at younger ages and BMI is a better measure of excess adiposity in young and middle-aged adults [48] than in the elderly [49]. This finding is consistent with many other reports in Whites [4], [8], [9], [18]–[21], but the variation in the BMI-mortality relationship by age had not been examined in Blacks.

The major strengths of this study are its large sample size, wide BMI range and long follow-up time. In addition, the large number of Black men and women in CPS-II allowed for the largest detailed analysis of the BMI-mortality relationship in Blacks to date. The main limitation of the study is the reliance on a single self-report of height and weight. When compared with measured height and weight, self-reported data systematically overestimate height in men and underestimate weight in women [50], although the magnitude of this error is small. A longitudinal study using national data collected over 20 years (1971–1992) showed that Black men aged 48–60 years had a higher average weight gain per year compared to White men (0.14 and 0.02 kg/year, respectively) [51]. In contrast, for women, especially Black women, weight gain tended to occur earlier in adulthood; thus, the single measure may more accurately reflect long-term weight in women and White men compared to Black men. In addition, it is also possible that exclusions for prevalent disease (cancer, heart disease, stroke, emphysema, chronic bronchitis or asthma) were not adequate to control for all disease-related weight loss.

In summary, this large nationwide study helps to clarify four major unresolved issues in the study of BMI and mortality by demonstrating that smoking and prevalent disease significantly modify the relationship between BMI and mortality, and that among never smokers without prevalent disease, overweight and obesity are strongly associated with subsequent risk of mortality and the optimal BMI range is 20.0–24.9 kg/m^2^. Additionally, this study demonstrated that among never smokers without prevalent disease, the BMI-mortality relationship is similar in Black and White men and women where overweight (BMI 25.0–29.9 kg/m^2^) is associated with a modest and obesity (BMI 30.0+ kg/m^2^) a more substantial increased risk of premature death. In the U.S., age-adjusted prevalence estimates of obesity are highest for African Americans (35.7%), followed by Hispanics (28.7%), and non-Hispanic Whites (23.7%) [52]. Given the high prevalence of obesity among all racial-ethnic populations, although disproportionately higher among African Americans, these findings are of considerable clinical and public health relevance.

## References

[1] Calle E, Rodriguez C, Walker-Thurmond K, Thun MJ (2003) Overweight, obesity, and mortality from cancer in a prospectively studied cohort of U.S. adults. N Engl J Med 348: 1625–1638. 12711737 10.1056/NEJMoa021423

[2] Whitlock G, Lewington S, Sherliker P, Clarke R, Emberson J, et al (2009) Body-mass index and cause-specific mortality in 900 000 adults: collaborative analyses of 57 prospective studies. Lancet 373: 1083–1096. 19299006 10.1016/S0140-6736(09)60318-4 PMC2662372

[3] Berrington de Gonzalez A, Hartge P, Cerhan JR, Flint AJ, Hannan L, et al (2010) Body-mass index and mortality among 1.46 million White adults. New England Journal of Medicine 363: 2211–2219. 21121834 10.1056/NEJMoa1000367 PMC3066051

[4] Calle EE, Thun MJ, Petrelli JM, Rodriguez C, Heath CW Jr (1999) Body-mass index and mortality in a prospective cohort of U.S. adults. N Engl J Med 341: 1097–1105. 10511607 10.1056/NEJM199910073411501

[5] Flegal KM, Kit BK, Orpana H, Graubard BI (2013) Association of all-cause mortality with overweight and obesity using standard body mass index categories: a systematic review and meta-analysis. JAMA 309: 71–82. 23280227 10.1001/jama.2012.113905 PMC4855514

[6] Zheng W, McLerran DF, Rolland B, Zhang X, Inoue M, et al (2011) Association between Body-Mass Index and Risk of Death in More Than 1 Million Asians. New England Journal of Medicine 364: 719–729. 21345101 10.1056/NEJMoa1010679 PMC4008249

[7] Stevens J (2000) Obesity and mortality in African-Americans. Nutrition Reviews 58: 346–358. 11140906 10.1111/j.1753-4887.2000.tb01832.x

[8] Adams KF, Schatzkin A, Harris TB, Kipnis V, Mouw T, et al (2006) Overweight, obesity, and mortality in a large prospective cohort of persons 50 to 71 years old. N Engl J Med 355: 763–778. 16926275 10.1056/NEJMoa055643

[9] McTigue K, Larson JC, Valoski A, Burke G, Kotchen J, et al (2006) Mortality and cardiac and vascular outcomes in extremely obese women. JAMA 296: 79–86. 16820550 10.1001/jama.296.1.79

[10] Reis JP, Araneta MR, Wingard DL, Macera CA, Lindsay SP, et al (2009) Overall obesity and abdominal adiposity as predictors of mortality in u.s. White and black adults. Ann Epidemiol 19: 134–142. 19185808 10.1016/j.annepidem.2008.10.008

[11] Boggs DA, Rosenberg L, Cozier YC, Wise LA, Coogan PF, et al (2011) General and abdominal obesity and risk of death among Black women. New England Journal of Medicine 365: 901–908. 21899451 10.1056/NEJMoa1104119 PMC3206314

[12] Stevens J, Keil J, Rust P, Tyroler H, Davis C, et al (1992) Body mass index and body girths as predictors of mortality in black and white women. Arch Intern Med 152: 1257–1262. 1599355

[13] Cornoni-Huntley JC, Harris TB, Everett DF, Albanes D, Micozzi MS, et al (1991) An overview of body weight of older persons, including the impact on mortality. J Clin Epidemiol 44: 743–753. 1941025 10.1016/0895-4356(91)90126-t

[14] Freedman D, WIlliamson D, Croft J, Byers T (1995) Relation of body fat distribution to ischemic heart disease. The National Health and Nutrition Examination Survey I Epidemiologic Follow-up Study. Am J Epidemiol 142: 53–63. 7785674 10.1093/oxfordjournals.aje.a117545

[15] Johnson H, Heineman E, Heiss GHC, Tyroler H (1986) Cardiovascular disease risk factors and mortality among black women and white women aged 40–69 years in Evans County, Georgia. Am J Epidemiol 123: 209–220. 3946371 10.1093/oxfordjournals.aje.a114230

[16] Stevens J, Plankey MW, Williamson DF, Thun MJ, Rust PF, et al (1998) The body mass index-mortality relationship in white and African American women. Obesity 6: 268–277. 10.1002/j.1550-8528.1998.tb00349.x 9688103

[17] Wienpahl J, Ragland DR, Sidney S (1990) Body mass index and 15-year mortality in a cohort of black men and women. J Clin Epidemiol 43: 949–960. 2213083 10.1016/0895-4356(90)90078-4

[18] Stevens J, Cai J, Pamuk ER, Williamson DF, Thun MJ, et al (1998) The effect of age on the association between body-mass index and mortality. N Engl J Med 338: 1–7. 9414324 10.1056/NEJM199801013380101

[19] Baik I, Ascherio A, Rimm EB, Giovannucci E, Spiegelman D, et al (2000) Adiposity and mortality in men. Am J Epidemiol 152: 264–271. 10933273 10.1093/aje/152.3.264

[20] Pischon T, Boeing H, Hoffmann K, Bergmann M, Schulze MB, et al (2008) General and abdominal adiposity and risk of death in Europe. N Engl J Med 359: 2105–2120. 19005195 10.1056/NEJMoa0801891

[21] Folsom AR, Kushi LH, Anderson KE, Mink PJ, Olson JE, et al (2000) Associations of general and abdominal obesity with multiple health outcomes in older women: the Iowa Women's Health Study. Arch Intern Med 160: 2117–2128. 10904454 10.1001/archinte.160.14.2117

[22] Garfinkel L (1985) Selection, follow-up, and analysis in the American Cancer Society prospective studies. National Cancer Institute monograph 67. Washington D.C.: Government Printing Office. pp. 49–52. 4047150

[23] Bulliten of the World Health Organization. 64: 929–941.

[24] Calle EE, Terrell DD (1993) Utility of the National Death Index for ascertainment of mortality among cancer prevention study II participants. Am J Epidemiol 137: 235–241. 8452128 10.1093/oxfordjournals.aje.a116664

[25] Freedman DM, Ron E, Ballard-Barbash R, Doody MM, Linet MS (2006) Body mass index and all-cause mortality in a nationwide US cohort. Int J Obes (Lond) 30: 822–829. 16404410 10.1038/sj.ijo.0803193

[26] Gelber RP, Kurth T, Manson JE, Buring JE, Gaziano JM (2007) Body mass index and mortality in men: evaluating the shape of the association. Int J Obes (Lond) 31: 1240–1247. 17342077 10.1038/sj.ijo.0803564

[27] Manson JE, Willett WC, Stampfer MJ, Colditz GA, Hunter DJ, et al (1995) Body weight and mortality among women. N Engl J Med 333: 677–685. 7637744 10.1056/NEJM199509143331101

[28] Singh PN, Lindsted KD (1998) Body mass and 26-year risk of mortality from specific diseases among women who never smoked. Epidemiology 9: 246–254. 9583415

[29] Bianchini F, Kaaks R, Vainio H (2002) Overweight, obesity, and cancer risk. Lancet Oncol 3: 565–574. 12217794 10.1016/s1470-2045(02)00849-5

[30] Koster A, Leitzmann MF, Schatzkin A, Adams KF, van Eijk JT, et al (2008) The combined relations of adiposity and smoking on mortality. Am J Clin Nutr 88: 1206–1212. 18996854 10.3945/ajcn.2008.26298 PMC2642004

[31] Ajani UA, Lotufo PA, Gaziano JM, Lee IM, Spelsberg A, et al (2004) Body mass index and mortality among US male physicians. Ann Epidemiol 14: 731–739. 15519894 10.1016/j.annepidem.2003.10.008

[32] Hjartaker A, Adami HO, Lund E, Weiderpass E (2005) Body mass index and mortality in a prospectively studied cohort of Scandinavian women: the women's lifestyle and health cohort study. Eur J Epidemiol 20: 747–754. 16170657 10.1007/s10654-005-2145-x

[33] Jain MG, Miller AB, Rohan TE, Rehm JT, Bondy SJ, et al (2005) Body mass index and mortality in women: follow-up of the Canadian National Breast Screening Study cohort. Int J Obes (Lond) 29: 792–797. 15809663 10.1038/sj.ijo.0802952

[34] Pednekar MS, Hakama M, Hebert JR, Gupta PC (2008) Association of body mass index with all-cause and cause-specific mortality: findings from a prospective cohort study in Mumbai (Bombay), India. Int J Epidemiol 37: 524–535. 18276634 10.1093/ije/dyn001

[35] Kuriyama S (2006) Impact of overweight and obesity on medical care costs, all-cause mortality, and the risk of cancer in Japan. J Epidemiol 16: 139–144. 16837764 10.2188/jea.16.139 PMC7603910

[36] Song YM, Ha M, Sung J (2007) Body mass index and mortality in middle-aged Korean women. Ann Epidemiol 17: 556–563. 17395488 10.1016/j.annepidem.2007.01.028

[37] Jee SH, Sull JW, Park J, Lee SY, Ohrr H, et al (2006) Body-mass index and mortality in Korean men and women. N Engl J Med 355: 779–787. 16926276 10.1056/NEJMoa054017

[38] Lawlor DA, Hart CL, Hole DJ, Davey Smith G (2006) Reverse causality and confounding and the associations of overweight and obesity with mortality. Obesity (Silver Spring) 14: 2294–2304. 17189558 10.1038/oby.2006.269

[39] Katzmarzyk PT, Craig CL, Bouchard C (2001) Original article underweight, overweight and obesity: relationships with mortality in the 13-year follow-up of the Canada Fitness Survey. J Clin Epidemiol 54: 916–920. 11520651 10.1016/s0895-4356(01)00356-0

[40] Lahmann PH, Lissner L, Gullberg B, Berglund G (2002) A prospective study of adiposity and all-cause mortality: the Malmo Diet and Cancer Study. Obes Res 10: 361–369. 12006635 10.1038/oby.2002.50

[41] Peeters A, Barendregt JJ, Willekens F, Mackenbach JP, Al Mamun A, et al (2003) Obesity in adulthood and its consequences for life expectancy: a life-table analysis. Ann Intern Med 138: 24–32. 12513041 10.7326/0003-4819-138-1-200301070-00008

[42] Janssen I (2007) Morbidity and mortality risk associated with an overweight BMI in older men and women. Obesity (Silver Spring) 15: 1827–1840. 17636102 10.1038/oby.2007.217

[43] Corrada MM, Kawas CH, Mozaffar F, Paganini-Hill A (2006) Association of body mass index and weight change with all-cause mortality in the elderly. Am J Epidemiol 163: 938–949. 16641311 10.1093/aje/kwj114 PMC3373260

[44] Ringback Weitoft G, Eliasson M, Rosen M (2008) Underweight, overweight and obesity as risk factors for mortality and hospitalization. Scand J Public Health 36: 169–176. 18519281 10.1177/1403494807085080

[45] Kivimaki M, Ferrie JE, Batty GD, Davey Smith G, Elovainio M, et al (2008) Optimal form of operationalizing BMI in relation to all-cause and cause-specific mortality: the original Whitehall study. Obesity (Silver Spring) 16: 1926–1932. 18551106 10.1038/oby.2008.322

[46] Flegal KM, Graubard BI, Williamson DF, Gail MH (2005) Excess deaths associated with underweight, overweight, and obesity. JAMA 293: 1861–1867. 15840860 10.1001/jama.293.15.1861

[47] Flicker L, McCaul K, Hankey G, Jamrozik K, Brown W, et al (2010) Body mass index and survival in men and women aged 70 to 75. J Am Geriatr Soc 58: 234–241. 20370857 10.1111/j.1532-5415.2009.02677.x

[48] Keys A, Fidanza F, Karvonen MJ, et al (1972) Indices of relative weight and obesity. J Chronic Dis 25: 329–343. 4650929 10.1016/0021-9681(72)90027-6

[49] Borkan G, Hults D, Gerzof S, Robbins A, Silbert C (1983) Age changes in body composition revealed by computed tomography. J Gerontol 38: 673–677. 6630900 10.1093/geronj/38.6.673

[50] Stevens J, Keil JE, Waid R, Gazes PC (1990) Accuracy of current, 4-year, and 28-year self-reported body weight in an elderly population. Am J Epidemiol 132: 1156–1163. 2260547 10.1093/oxfordjournals.aje.a115758

[51] Sheehan TJ, DuBrava S, DeChello LM, Fang Z (2003) Rates of weight change for black and white Americans over a twenty year period. Int J Obes 27: 498–504. 10.1038/sj.ijo.0802263 12664083

[52] MMWR. 58: 740–744.

